# How Cold Is Cold Enough? Refrigeration of the Next-Generation Impactor to Prevent Aerosol Undersizing

**DOI:** 10.1089/jamp.2021.0015

**Published:** 2022-02-14

**Authors:** Uwe Schuschnig, Benjamin Heine, Martin Knoch

**Affiliations:** PARI Pharma GmbH, Gräfelfing, Germany.

**Keywords:** droplet size determination, impactor cooling, next generation impactor

## Abstract

***Background:*** Heat transfer from impactor to aqueous aerosols causes underestimation of droplet size due to evaporation. Hence, pharmacopeia suggests cooling the impactor to 5°C, which is well below aerosol temperature. In this study, we assessed the droplet size at four different impactor temperatures under controlled ambient conditions to compare the compendial 5°C method with our in-house method, where the impactor is cooled to aerosol temperature.

***Materials and Methods:*** A single nebulizer/compressor unit was used throughout. It produced an aerosol at 17°C when operated at 50% RH and 23°C RT ambient conditions. Thirty-six experiments were conducted with saline, 9 each at impactor temperatures of 5°C, 10°C, 17°C, and 23°C. NaCl stage deposition was determined by conductometry, mass on stages by weighing. Moreover, a simulation was carried out to track aerosol temperature when entering the impactor.

***Results:*** Measuring at 23°C yields a significantly smaller mass median aerodynamic diameter (MMAD) than at 5°C–17°C. Despite elevated water condensation in the impactor at 5°C and 10°C, there was no increase in MMAD compared with 17°C. Instead, droplet size determination at 5°C led to significantly smaller values than at 17°C, probably due to distorted volumetric impactor flow rates at different impactor temperatures. Reevaluation of data with flow rates adjusted for impactor temperature (14.1 L/min at 5°C vs. 15.0 L/min at 23°C) led to indistinguishable results at 5°C–17°C. A computational fluid dynamics (CFD) simulation confirmed rapid cooling of the incoming air within the inlet and stage 1 and, with it, the systematic droplet undersizing due to reduced volumetric airflow using a cooled impactor.

***Conclusions:*** As long as the impactor temperature is at or below aerosol temperature, no effects on droplet size can be observed. Measuring at aerosol temperature yields the same results as at 5°C, but prevents condensation. However, cooling the impactor well below ambient temperature can cause a systematic error in the volumetric flow rate through the impactor if not corrected accordingly.

## Introduction

Aqueous droplets are affected by multiple factors,^(1,2)^ and the method used for measuring droplet size can have a considerable impact on the result.

For cascade impactor measurements, it was shown that the impactor temperature has a significant impact on droplet size. An impactor at ambient temperature heats the relatively cold aerosol, which results in droplet shrinkage due to humidification of the air by evaporation from the droplets.^(3,4)^ The authors also demonstrated that this systematic error could be eliminated by cooling the impactor to aerosol temperature in a water bath. These findings were confirmed for a number of different jet nebulizers^(5)^ by showing that the mass median aerodynamic diameter (MMAD) of a noncooled impactor is only 40%–80% of the value obtained in a cooled impactor.

Aerosol undersizing could also be prevented by using 100% RH air to operate the nebulizer or by heating the nebulizer to prevent cooling of the aerosol.^(2)^ However, these methods have an impact on nebulizer performance and consequently alter droplet size at the mouthpiece. Hence, cooling the impactor remains the only viable option to obtain realistic droplet size characterization of aqueous aerosols through cascade impaction.

The next-generation impactor (NGI), initially designed by V.A. Marple for characterization of metered dose inhalers (MDIs) and dry powder inhalers (DPIs),^(6)^ which does not require refrigeration, was later calibrated for lower flow rates, making it suitable for nebulizer testing.^(7)^ Due to its user-friendly design and the more physiological flow rate, the NGI superseded the Andersen cascade impactor for pharmaceutical nebulizer testing. However, with its wide design for easy assembly and operation, it became difficult to immerse these impactors in a standard laboratory water bath for temperature control.

Hence, a method based on impactor refrigeration was proposed to circumvent cooling in a water bath.^(8)^ According to this method, the impactor is cooled for 90–120 minutes in a refrigerator set to 5°C. Then, the impactor is placed on the laboratory bench and the experiment is started within 5 minutes of removal from the refrigerator. The authors demonstrated this to be a robust, reproducible, and realistic method of reflecting the characteristics of the aerosol leaving the mouthpiece of the nebulizer.

Consequently, this method was further evaluated in a multicenter study^(9)^ and proposed by the European Pharmaceutical Aerosol Group for the droplet size analysis of nebulizer-produced aerosols for the European Pharmacopoeia general chapter 2.9.44 covering preparations for nebulization. Later, this method was also suggested by the USP<1601>.

Even though impactor chilling in the refrigerator enables realistic measurements with standard readily available materials, this method also has several downsides.

First, it requires at least 90 minutes of cooling in the refrigerator, limiting the number of experiments per impactor to about 1 every 2 hours, when strictly following instructions.^(8,9)^ Second, as the impactor is much colder than the incoming aerosol, water condensation on the impactor walls necessitates intensive cleaning and drying postexperiment, leading to reheating of the device. Third, at the end of the day, the entire impactor has to be completely disassembled and thoroughly dried to prevent corrosion.^(9)^ All these factors make impactor measurements more labor-intensive and require multiple impactors to increase the number of experiments per day.

An alternative method is to cool the impactor to aerosol temperature in a cooling cabinet, which is commercially available. This approach offers several advantages over separate chilling to 5°C in a refrigerator. First and most importantly, the rate of experiments increases since the impactor remains cool in the impactor cooler and only the stage trays and inlets are exchanged after the experiments. This allows a higher frequency of experiments per impactor at reduced labor intensity. Second, the impactor temperature remains constant during an experiment as the impactor is not warmed by the environment or aerosol, potentially reducing MMAD variations. Third, there is less effort needed to protect the impactor from corrosion.

In this study, we investigated the effect of the impactor temperature on droplet size with a single breath-enhanced jet nebulizer, comparing the compendial 5°C method with impactor temperatures of 10°C, 17°C, and 23°C. The study was supported using a computational fluid dynamics (CFD) simulation focusing on the temperature distribution upstream of stage 1.

Furthermore, we present a fast and easy conductometric method to determine the aerosol mass on the stages and compare this with a validated internal standard high-performance liquid chromatography (HPLC) method using salbutamol.

## Materials and Methods

A single nebulizer/compressor unit (PARI LC SPRINT STAR/BOY SX) was used for all experiments to minimize device variability in the core investigation of NGI temperature effects. All experiments were performed in a laboratory with well-controlled ambient conditions of 23°C ± 1°C and 50% ± 5% RH.

The LC SPRINT STAR was expected to be most susceptible to impactor temperature (from the SPRINT nebulizers) as it produces the smallest particles at the lowest output rate, facilitating particle shrinkage through evaporation.

Even though the LC SPRINT STAR still has a relatively high output rate (i.e., produces a high concentration of aerosol) compared with nonbreath-enhanced nebulizers (e.g., T-piece nebulizers), the main observations are expected to transfer to other nebulizer systems as well.

### Aerosol temperature measurement

The nebulizer was filled with 5 mL of isotonic saline and connected using a T-piece and filter to a vacuum source drawing air at 15 L/min through the assembly. A Pt100 resistance temperature detector (RTD) (Greisinger GMH 3700) with 3 mm diameter was tightly inserted using the T-piece into the aerosol stream. The temperature readings stabilized at 17°C after about 4 minutes.

### Impactor temperature measurement

A small aluminum block with holes accommodating the temperature sensors of the cooling cabinet and the Pt100 RTD was attached under the top handle of the NGI. The aluminum block was insulated (by a 3D printed plastic cover) from the circulating air stream within the cabinet.

### Impactor cooling

We used a cooling cabinet manufactured by Künzel & Sohn similar to commercially available NGI coolers. The cooler is equipped with a temperature controller (Dixell cool mate), thus enabling the temperature of the impactor to be kept in a range of ±0.5°C. The impactor temperature reading from the RTD was noted at the beginning and end of each experiment.

### Impactor testing

The volumetric impactor (COPLEY) flow rate was set to 15 L/min with a manual flow controller (BROOKS FCA8902) between the impactor outlet and vacuum source. The correct flow rate was verified before each experiment with a calibrated flow meter (TSI 4040 H) connected to the impactor inlet. The dry weight of the stages was determined.

The nebulizer was filled with 4 mL of 0.9% NaCl solution and tightly fitted to the NGI inlet using a rubber connector (Intersurgical). Then, the nebulizer was operated for 5 minutes with a PARI BOY SX compressor.

At the end of the experiment, the stages were weighed and the sodium chloride content subsequently determined.

### Determination of NaCl amounts through conductometry

NaCl deposits were extracted from all stages as well as the inlet, each with 10 mL of purified water. The water was then transferred to 50-mL centrifuge tubes (Falcon) and the conductometer probe (Lovibond SD 320 Con) was placed in the tube. The reading was recorded after stabilization and the probe rinsed in pure water before placing it in the next tube.

### Data evaluation and cutoff size calculation

Since the COPLEY CITAS software does not calculate at flow rates below 15 L/min, we used an Excel spreadsheet to calculate the MMAD and cutoff sizes. We used the equation *D*_50,2_ = *D*_50,1_ × SQR(*Q*_1_/*Q*_2_)^(10)^ and the 15-L/min cutoff sizes, as in the publication.^(7)^ Data evaluation was performed using Statgraphics Centurion 17 software.

### Comparison with the salbutamol internal standard HPLC method

A validated, in-house test method based on an internal standard (5 μg/mL bamethane sulfate in purified water) was used in a separate set of experiments for comparison with the conductometry method. The conductivity meter was calibrated with standards prepared from a salbutamol inhalation solution (Ventolin, 0.1% salbutamol, and 0.9% NaCl in water) and internal standard solution. Six NGI experiments were carried out at impactor temperature of 17°C with three different nebulizers (LC SPRINT STAR, LC SPRINT, and LC SPRINT JUNIOR) and two different compressors (1.0 and 1.6 bar) using Ventolin. The assay of the resulting samples was analyzed by HPLC and conductometry.

### Simulation of the conjugate heat transfer

To estimate the temperature development in the air stream, an unsteady 3D-RANS-CFD simulation of the upstream section of the impactor, including throat and stage 1, was performed. The computational mesh was generated using hexahedral and prism cells, yielding a fine mesh with 200,000 cells in the solid domain and 550,000 cells in the fluid domain. Humidity was modeled using a multigas approach of air and water vapor and condensation using a fluid film approach. Heat transfer from fluid to solid and from solid to the environment was enabled. Physical simulation time was selected in accordance with the experiment (300 seconds) as well as the boundary conditions: air temperature at the inlet (downstream of nebulizer) was 17°C at a relative humidity of 100%. Temperatures of the impactor wall and the environment were 5°C, 10°C, and 17°C. Reference laboratory conditions were 23°C at a flow rate of 15 L/min.

## Results

### Comparison of HPLC with conductometry

**FIG. 1.**
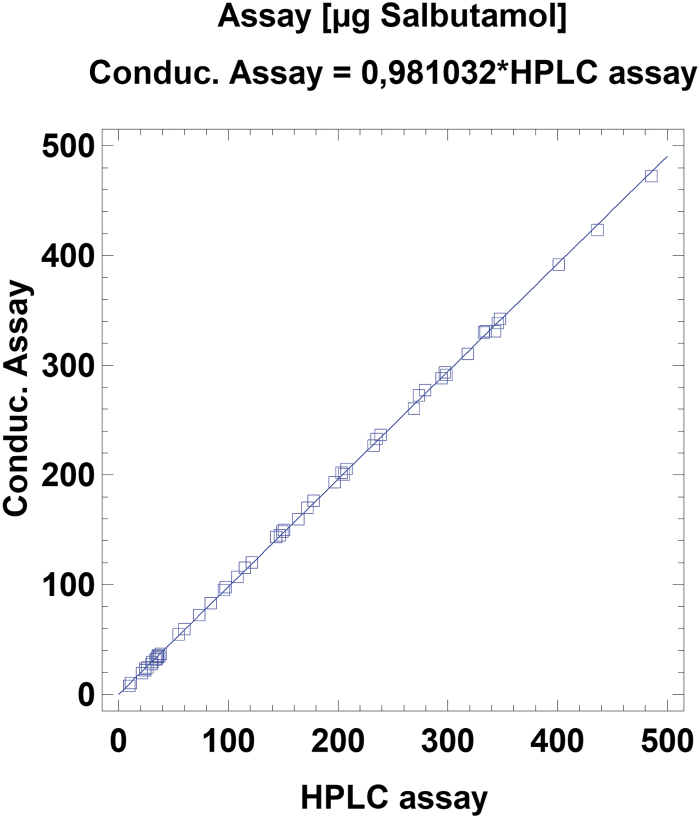
Plot of salbutamol assay measured by HPLC versus conductometric determination. *R*^2^ = 0.99898. HPLC, high-performance liquid chromatography.

**FIG. 2.**
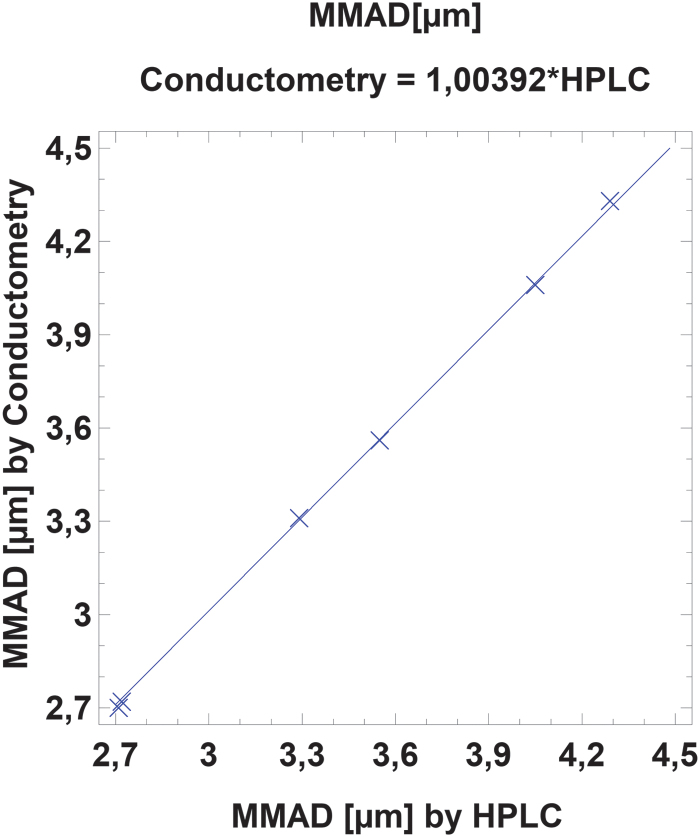
Plot of MMAD (μm) determined by HPLC versus conductometric measurement. *R*^2^ = 0.999984. MMAD, mass median aerodynamic diameter.

Both analytic methods showed very high correlations of *R*^2^ > 0.99 (Figs. 1 and 2). There was also no significant difference between the results obtained by HPLC or conductometry (*p* > 0.90 for assay and *p* > 0.97 for MMAD).

### Mass on stages

**FIG. 3.**
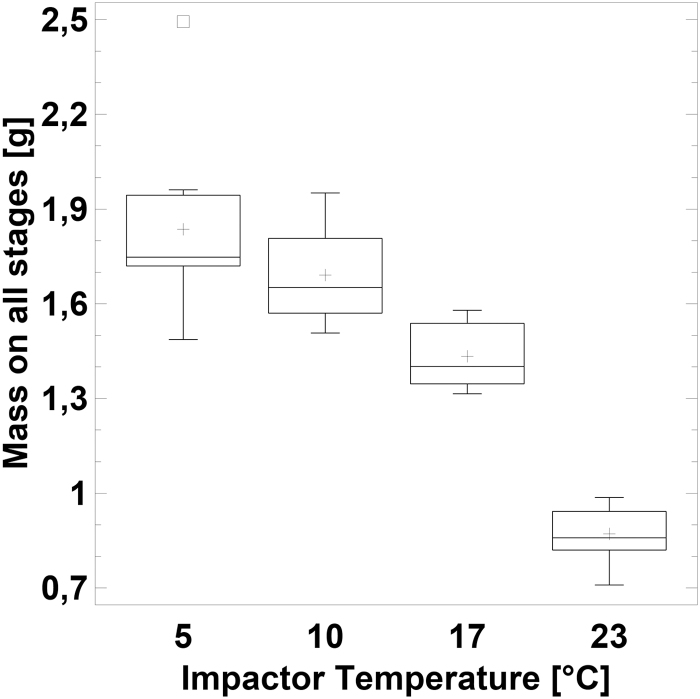
Liquid mass on impactor stages determined gravimetrically versus impactor temperature.

The mass of liquid found on all impactor stages was 0.87 ± 0.03 g at 23°C, 1.43 ± 0.4 g at 17°C, 1.69 ± 0.05 g at 10°C, and 1.84 ± 0.10 g at 5°C. Comparing the weighed values versus the analytically determined mass, 61% of the analytic mass was found at 23°C, 98% at 17°C, 114% at 10°C, and 131% at 5°C, indicating increasing water condensation with decreasing impactor temperature (Table 1 and Fig. 3).

**Table 1.** Tabulated Results and Statistics from Stage Weighing; Values Are Liquid Mass in Grams

Set temperature [°C]	Count	Average mass [g]	Standard deviation	Coefficient of variation	Minimum	Maximum	Range
23	9	0.870733	0.0896928	10.3008%	0.709	0.986	0.277
17	8	1.43313	0.104446	7.28799%	1.315	1.579	0.264
10	9	1.69133	0.151657	8.96669%	1.507	1.951	0.444
5	9	1.83578	0.29146	15.8767%	1.487	2.494	1.007

Visually, condensation appeared to be highest at the impactor inlet and the first impactor stage.

### MMAD at 15 L/min

**FIG. 4.**
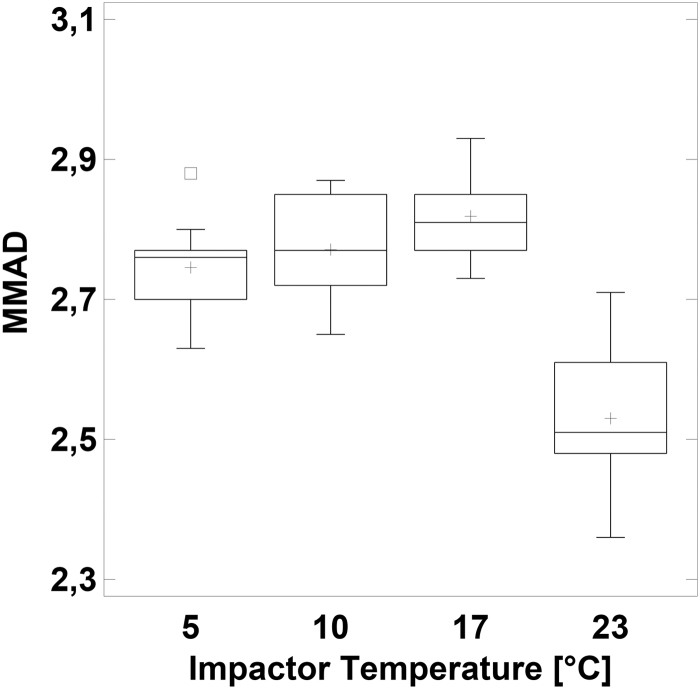
Box and whisker plot of the MMAD (μm) determined at the nominal flow rate of 15 L/min versus impactor temperature.

All droplet sizes measured at or below the aerosol temperature of 17°C were significantly higher than at room temperature (23°C) (*p* < 0.05). The MMAD seemed to be highest at 17°C and was significantly higher than the MMAD measured at impactor temperature of 5°C (*p* = 0.044) (Table 2 and Fig. 4).

**Table 2.** Summary Statistics for Mass Median Aerodynamic Diameter [μm]

Set temperature [°C]	Count	Average MMAD [μm]	Standard deviation	Coefficient of variation	Minimum	Maximum	Range
5	9	2.74556	0.072476	2.63976%	2.63	2.88	0.25
10	9	2.77111	0.079285	2.86113%	2.65	2.87	0.22
17	9	2.81889	0.0702575	2.49238%	2.73	2.93	0.2
23	9	2.53	0.106771	4.22019%	2.36	2.71	0.35

MMAD, mass median aerodynamic diameter.

### MMAD at corrected flow rate

**FIG. 5.**
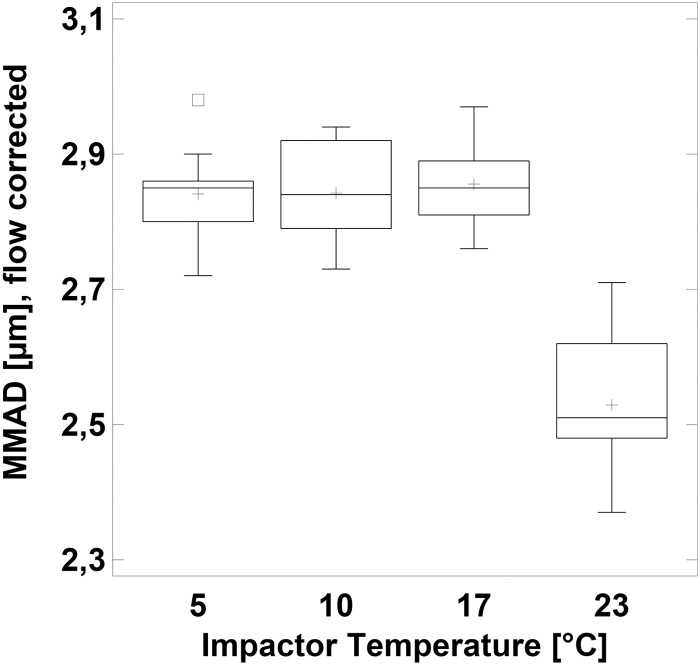
Box and whisker plot of the MMAD (μm) determined at temperature-corrected flow rate versus impactor temperature.

When the cutoff sizes of the impactor stages are calculated for flow rates obtained from the ideal gas law at the respective temperatures (14.1 L/min at 5°C; 14.3 L/min at 10°C; 14.7 L/min at 17°C; and 15.0 L/min at 23°C), the MMADs at 5°C, 10°C, and 17°C are indistinguishable (*p* = 0.9) (Table 3 and Fig. 5).

**Table 3.** Summary Statistics for Mass Median Aerodynamic Diameter [μm] with Temperature-Corrected Impactor Flow Rate

Set temperature [°C]	Count	Average MMAD [μm]	Standard deviation	Coefficient of variation	Minimum	Maximum	Range
5	9	2.84111	0.0742369	2.61295%	2.72	2.98	0.26
10	9	2.84222	0.0774238	2.72406%	2.73	2.94	0.21
17	9	2.85556	0.072476	2.53807%	2.76	2.97	0.21
23	9	2.52889	0.104934	4.1494%	2.37	2.71	0.34
Total	36	2.76694	0.160656	5.80627%	2.37	2.98	0.61

### Results of the conjugate heat transfer simulation

**FIG. 6.**
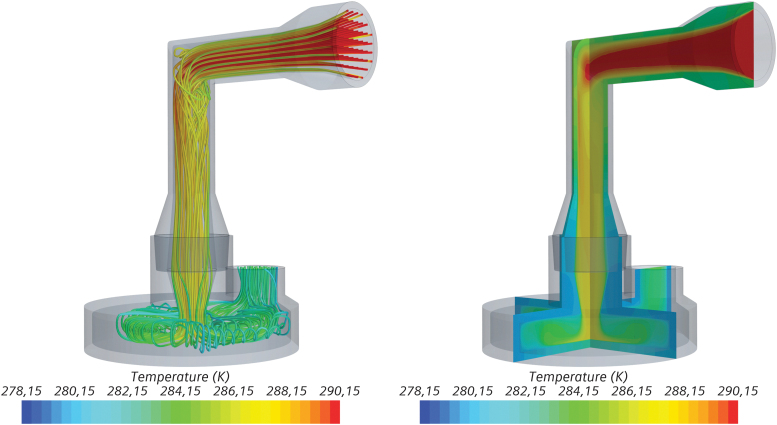
Temperature distribution in the upstream section of the impactor visualized by streamlines (left) and slices (right) 150 seconds after the start of the simulation; impactor cooled to 5°C.

As a result of expansion of compressed air and evaporation, air temperature drops from 23°C to ∼17°C as it is guided through the jet nebulizer. When the saturated air enters the impactor, the temperature reduces rapidly by forced convection at the impactor walls if they are colder than 17°C (Fig. 6).

Following the ideal gas law, the increase in density is inversely proportional to temperature. Considering mass conservation, velocity and volume flow rate decrease proportionally to temperature. Temperature, air density, and volume flow rate downstream of stage 1 extracted from the simulation are summarized in Table 4.

**Table 4.** Influence of Impactor Temperature on Air Temperature, Density, and Volume Flow Rate Downstream of Stage 1

Set impactor temperature	Time in experiment	Temperature downstream stage 1 [K]	Density downstream stage 1 [kg/m^3^]	Volume flow rate downstream stage 1 [L/min]	Deviation to set the volume flow rate [%]
5°C/278.15 K	Start	280.72	1.2520	14.07	6.2
	End	283.06	1.2408	14.21	5.3
	Average	282.00	1.2459	14.14	5.7
10°C/283.15 K	Start	284.65	1.2334	14.30	4.7
	End	286.27	1.2257	14.41	3.9
	Average	285.53	1.2292	14.36	4.3
17°C/290.15 K (reference)		290.15	1.2000	14.7	2.0

Reference at nebulizer inlet: 15 L/min at 23°C.

The results of the simulation confirm the assumption made for the MMAD measurements, that is, temperature is quickly reduced to near impactor temperature. Downstream of stage 1, the air temperature has already reached 80% of the temperature difference between inlet and impactor temperatures. Consequently, at downstream stage 1, the volume flow rate is up to 5.7% lower than what was set. In the following stages, temperature will asymptotically approach the impactor temperature.

## Discussion

When the study was planned, we expected to find increasing droplet sizes as water vapor condensation increases with colder impactor temperatures.

While the mass on the stages increased with lower temperatures, as expected, with about 30% mass from condensation at 5°C, there was no further increase in droplet size. Hence, condensation seems to occur exclusively on hypothermic surfaces and not at the droplets. The droplet temperature seemingly follows the air temperature without measurable particle growth.

Surprisingly, we found that the MMAD peaks at an impactor temperature equal to the aerosol temperature of 17°C and declined with both colder and warmer impactor temperatures (Fig. 4). While smaller droplet sizes at higher impactor temperatures are well understood and described in the literature,^(1–5)^ finding an explanation for the slightly, but significantly, smaller droplet sizes at 5°C was less easy.

We hypothesized that this finding was due to changes in volumetric flow rate through the impactor, leading to changes in the cutoff diameters. We assume that the incoming aerosol is rapidly cooled to impactor temperature and, thus, volume flow rate effectively decreases along the stages. This allows calculation of the temperature-driven change in airflow rate according to the ideal gas law, and the corresponding stage cutoff diameters can be derived therefrom.

**FIG. 7.**
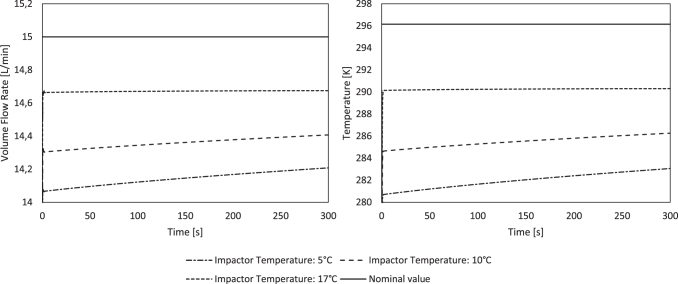
Temperature (right) and volume flow rate (left) development downstream of stage 1 as a function of time for three impactor temperatures and the nominal value.

Using this approach, the MMAD values recalculated with the corrected flow rates (14.1 L/min at 5°C; 14.3 L/min at 10°C; and 14.7 L/min at 17°C vs. 15.0 L/min at 23°C) and droplet diameters were virtually identical at 5°C, 10°C, and 17°C (Fig. 5), confirming our hypothesis. Hence, if the impactor is cooled to 5°C and the ambient temperature is 23°C, the flow rate at the inlet should be set to ∼16 L/min to achieve 15 L/min through the impactor stages. To further corroborate the assumption that the incoming air stream is rapidly cooled to impactor temperature, a CFD study was conducted (Fig. 6). At the beginning of the experiment, air is indeed rapidly cooled down: the incoming air at 17°C leaves the first stage at only 7.6°C at an impactor temperature of 5°C (Table 4). Due to condensation and forced convection at the inner walls of the impactor, heat is transferred, leading to rising temperatures of the impactor over the duration of the experiment. Therefore, the temperature of air at the end of the experiment after 300 seconds is 2.3°C higher than at the beginning, leading to a slightly smaller temperature difference (Fig. 7 right).

Hence, using an impactor precooled at 5°C, during the experiment, along with the temperature, the volume flow rate within the impactor also rises (approx. +1%) (Fig. 7 left). This leads to a slight distortion of the cutoff diameters during measurement and the calculated MMAD values. In addition, the temperature shift may cause a slightly higher geometric standard deviation of the droplet size distribution. However, probably due to the very small volume flow error of 1%, we could not find a statistically significant difference in the experimental results.

While our general finding of smaller droplet sizes when using a noncooled impactor is consistent with published literature,^(3–5)^ the difference we found is far less pronounced. We found only a difference of 11% in MMAD, while other authors stated 20%–60%. In addition, others reported aerosol temperatures of 10°C exiting the nebulizer, while we measured 17°C. We believe that these differences are, although using different nebulizers, primarily due to different methodologies. The earlier studies^(3–5)^ used the Andersen cascade impactor, which is operated at almost twice the flow rate of the NGI (28.3 L/min vs. 15 L/min). Moreover, the authors performed their tests at lower ambient relative humidity (36%)^(4)^ and operated the nebulizers with dry compressed air,^(3,4)^ both factors enabling more water evaporation compared with 50% RH and compressor operation, as in this study. Probably, all these differences contribute to the deviations between our data and those published earlier.

The effect of impactor temperature on droplet size depends also on the nebulizer used. Nebulizers with a high aerosol output rate, yielding high aerosol concentrations, are less prone to particle shrinkage. For a vibrating membrane nebulizer with a high output rate (e.g., eFlow), we found only a difference of 0.2 μm in droplet size at the impactor temperature of 13°C versus 23°C.^(11)^ Other authors^(5)^ also reported higher differences for low-output T-piece nebulizers than for breath-enhanced nebulizers, which generate higher aerosol concentrations.

Conditioning the impactor to 5°C or 10°C requires about 2–3 hours in the impactor cooler, which prevented a randomization of the study. Hence, we included the order of experiment as a covariate in the statistical analysis, but could not observe an effect on droplet size.

MMAD measurement upon conductometric determination of NaCl correlated very well with the validated salbutamol HPLC analysis, with only 2% difference in the sample assay and 0.4% difference in MMAD. Conductometry yielded 98% of the HPLC method, probably because samples were diluted with deposited and condensed water on the stages and with purified water residues on the probe from rinsing. While the internal standard of the HPLC method compensates for these slight volumetric errors, the conductometric method does not. However, MMAD results were almost identical for both methods, and conductometry proved to be a suitable, quick, and easy method to conduct surrogate measurements for device testing as isotonic saline behaves like many commercially available inhalation solutions. Testing with isotonic saline also has the advantage of being nontoxic and does not require safety measures such as an extraction hood.

The core finding of our study is that once the impactor is cooled to aerosol temperature or below, there is no further significant change in droplet size. However, an impactor temperature of 5°C (or 10°C) necessitates that correct flow rates are used, either for operation of the impactor or for calculation of the cutoff diameters.

Conducting impactor testing at the temperature of the aerosol exiting the nebulizer in a special impactor cooler offers a number of advantages over the compendial method. Most importantly, the rate of experiments per impactor increases since the impactor remains cool in the impactor cooler and only the stage trays and inlets are exchanged. Hence, fewer impactors are needed for routine measurements. As the price of a cooling cabinet is only a quarter of that of the impactor, the required time for a return on investment is much shorter. In addition, maintenance of the cooling cabinet is less laborious than having a huge number of NGIs mensurated every year.

We also expect that working with a constantly cooled impactor at aerosol temperature reduces variations in results, while a refrigerated impactor is continuously warming at room temperature, leading to flow rate variations during the experiment.

Last, but not least, measuring at aerosol temperature minimizes condensation as well as reduces cleaning and drying efforts. Furthermore, it reduces the potential for corrosion.

## Notes

Reviewed by:

Warren Finley

John Dennis
